# Supplementary material for: Haplotype variability in mitochondrial rRNA predisposes to metabolic syndrome
Source: Commun Biol. 2024 Sep 11;7:1116. doi: 10.1038/s42003-024-06819-w (PMC11391015; doi:10.1038/s42003-024-06819-w)
Supplement: Supplementary file 2 — Supplementary Material [file 42003_2024_6819_MOESM2_ESM.pdf]

Supplementary Material

Manuscript title:

Haplotype variability in mitochondrial rRNA predisposes to metabolic syndrome

Authors:

Petr Pecina[1], Kristýna Čunátová[1], Vilma Kaplanová[1], Guillermo Puertas-Frias[1], Jan Šilhavý[2], Kateřina Tauchmannová[1], Marek Vrbacký[1], Tomáš Čajka[3], Ondřej Gahura[4], Markéta Hlaváčková[5], Viktor Stránecký[6], Stanislav Kmoch[6], Michal Pravenec[2], Josef Houštěk[1], Tomáš Mráček[1,*] and Alena Pecinová[1,*]

Contents:

Supplementary Tables 1-3

Supplementary Figures 1-9

Supplementary table 1. Amino acid (AA) substitutions caused by mitochondrial DNA variants in mtSHR, mtF344 and mtBN strains.

OXPHOS gene	AA No.	mtSHR	mtF344	mtBN
mt-Nd2	18	Ala	Val	Val
	150	Ser	Asn	Asn
	244	Ala	Ala	Thr
	265	Thr	Ala	Ala
	304	Met	Thr	Met
	316	His	1AA del	His
mt-Nd4	23	Thr	Ile	Ile
	356	Thr	Ala	Ala
	393	Ile	Met	Ile
	401	Ile	Val	Val
	405	Met	Met	Ile
mt-Nd6	30	Phe	Phe	Leu
	139	Val	Ile	Ile
mt-Cytb	214	Asn	Asp	Asp
	334	Val	Val	Ile
mt-Co1	2	Phe	Phe	Leu
	406	Asn	Asp	Asn
mt-Co2	165	Val	Ile	Val
mt-Atp6	35	Lys	Glu	Glu
mt-Atp8	58	Ile	Ile	Thr

Supplementary table 2. Mitochondrial DNA variants in transfer and ribosomal RNA genes in mtF344 and mtBN strains compared to mtSHR. Nucleotide No.: position in mtSHR (genome numbering). NC – no change in sequence with respect to mtSHR sequence.

	gene	Nucleotide No.	mtF344	mtBN
mt-tRNA	*Phe*	m.32A	del A	del A
		m.52C	ins A	ins A
	Cys	m.5198G	G>A	G>A
		m.5200G	G>A	G>A
		m.5235A	A>T	A>T
	Tyr	m.5267C	C>G	C>G
	Asp	m.6976A	A>G	A>G
	His	m.11540A	NC	A>G
	Thr	m.15331A	A>G	NC
	Pro	m.15398C	NC	C>T
mt-rRNA	*mt-Rnr1*	m.648C	NC	C>A
		m.935G	G>A	NC
		m.942T	T>C	NC
	mt-Rnr2	m.1099C	NC	C>T
		m.1132C	del C	NC
		m.1137C	C>A	C>A
		m.1223G	G>A	NC
		m.1248T	T>C	NC
		m.1521A	A>G	A>G
		m.1585C	C>T	C>T
		m.1653T	ins AC	NC
		m.1693T	NC	T>C
		m.1716C	C>T	C>T
		m.1832A	A>G	NC
		m.1918A	NC	G>A
		m.2170C	C>T	NC
		m.2647T	NC	del T

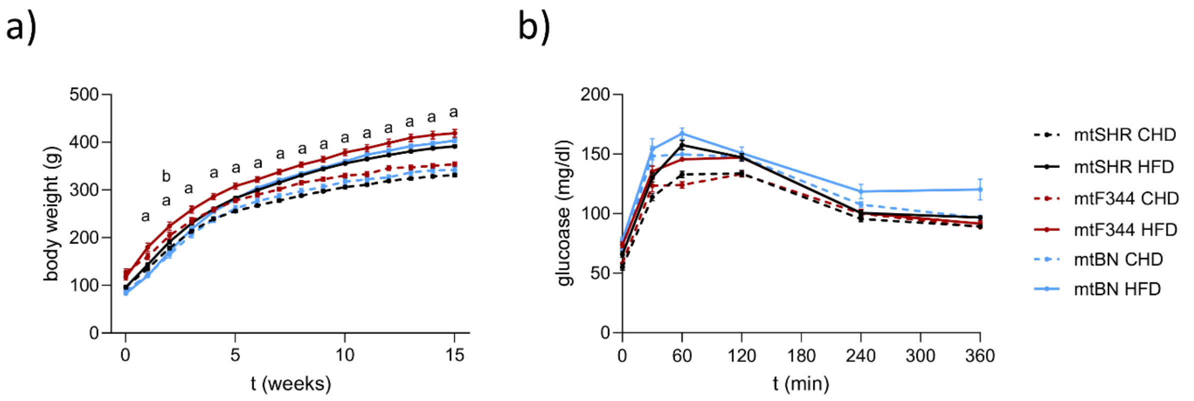

Supplementary figure 1: Metabolic phenotype of the conplastic strains. (**a**) Body weights of mtSHR, mtBN and mtF344 animals during 15 weeks on chow (CHD) or high-fat (HFD) diet. The significant change between mtF344 and mtSHR on HFD diet is depicted as *a* and between mtBN and mtSHR as *b* (p-value < 0.05) (**b**) Time course of glycemia during oral glucose tolerance test (OGTT). Data represents means ± S.D. from at least 10 animals.

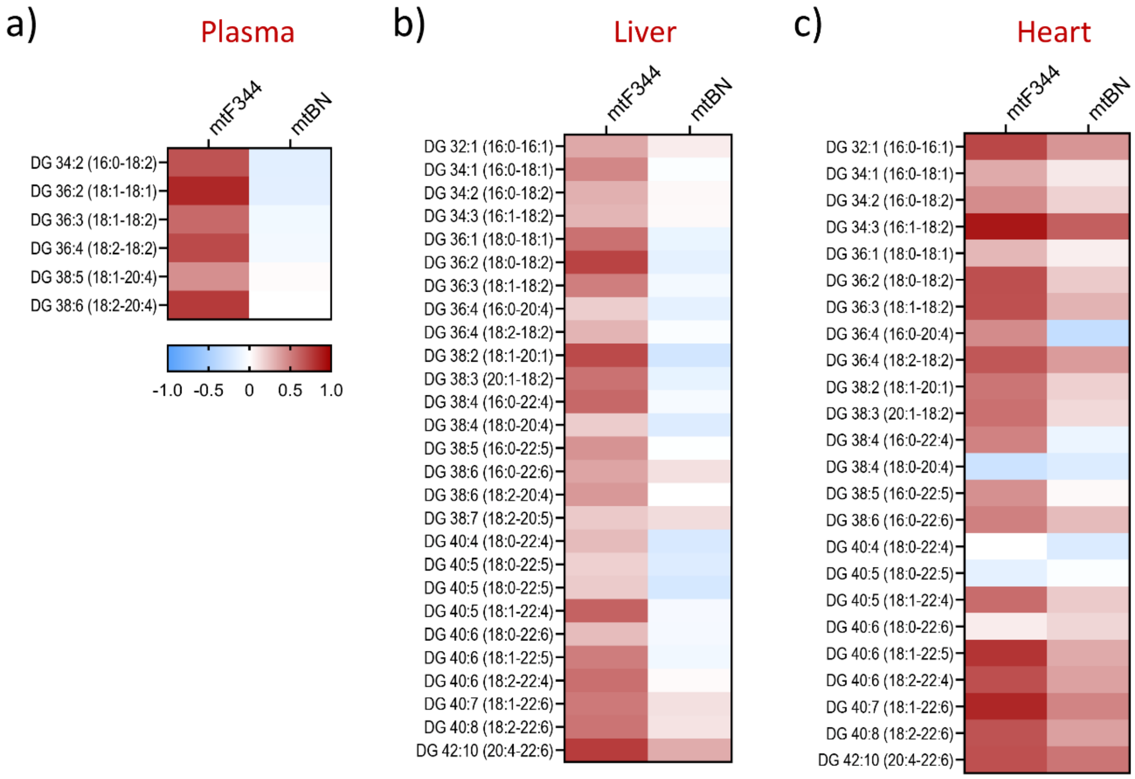

Supplementary figure 2: LC-MS analysis of individual diacylglycerols in the conplastic strains on HFD. The heatmap represents average $\log_2$ fold change of individual diacylglycerols compared to mtSHR control in plasma (**a**), liver (**b**), and heart (**c**) of mtF344 and mtBN strains (n = 6).

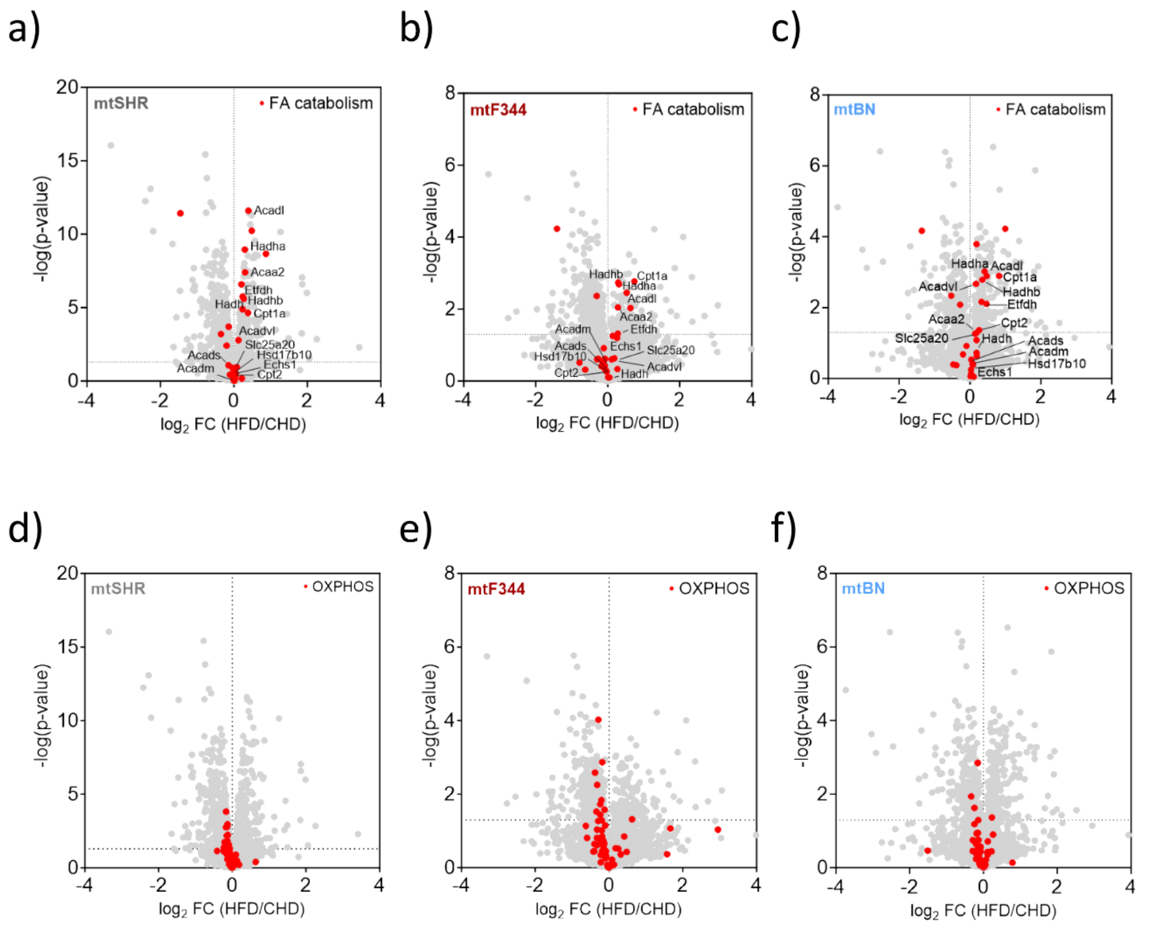

Supplementary figure 3: Label-free quantification mass spectrometry analysis (LFQ-MS) of liver tissue. Volcano plots depict differential contents of all proteins in between HFD and CHD in mtSHR (**a, d**), mtF344 (**b, e**) and mtBN (**c, f**). Red circles represent proteins involved in fatty acid (FA) degradation (**a-c**) and OXPHOS (**d-f**) according to KEGG pathways, enzymes of mitochondrial β-oxidation are labeled. Each point represents the data from 6 animals.

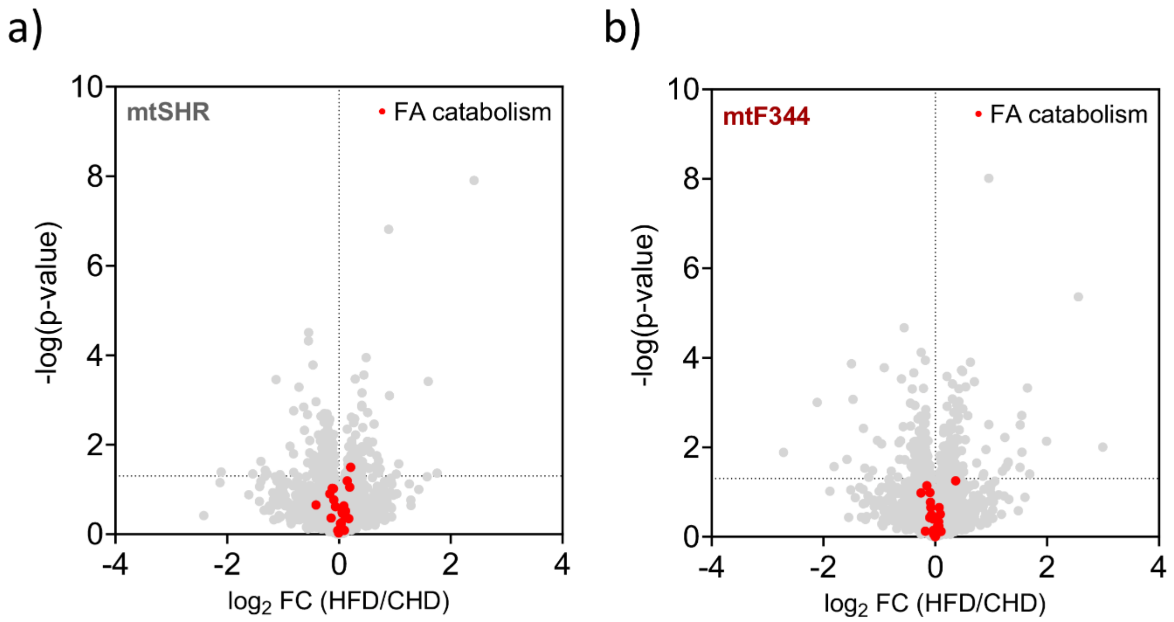

Supplementary figure 4: Label-free quantification mass spectrometry analysis (LFQ-MS) of heart tissue. Volcano plots depict differential contents of all proteins in between HFD and CHD in mtSHR (**a**) and mtF344 (**b**) strains. Red circles represent proteins involved in fatty acid (FA) degradation (according to KEGG pathways), enzymes of mitochondrial β-oxidation are labeled. Each point represents the data from 6 animals.

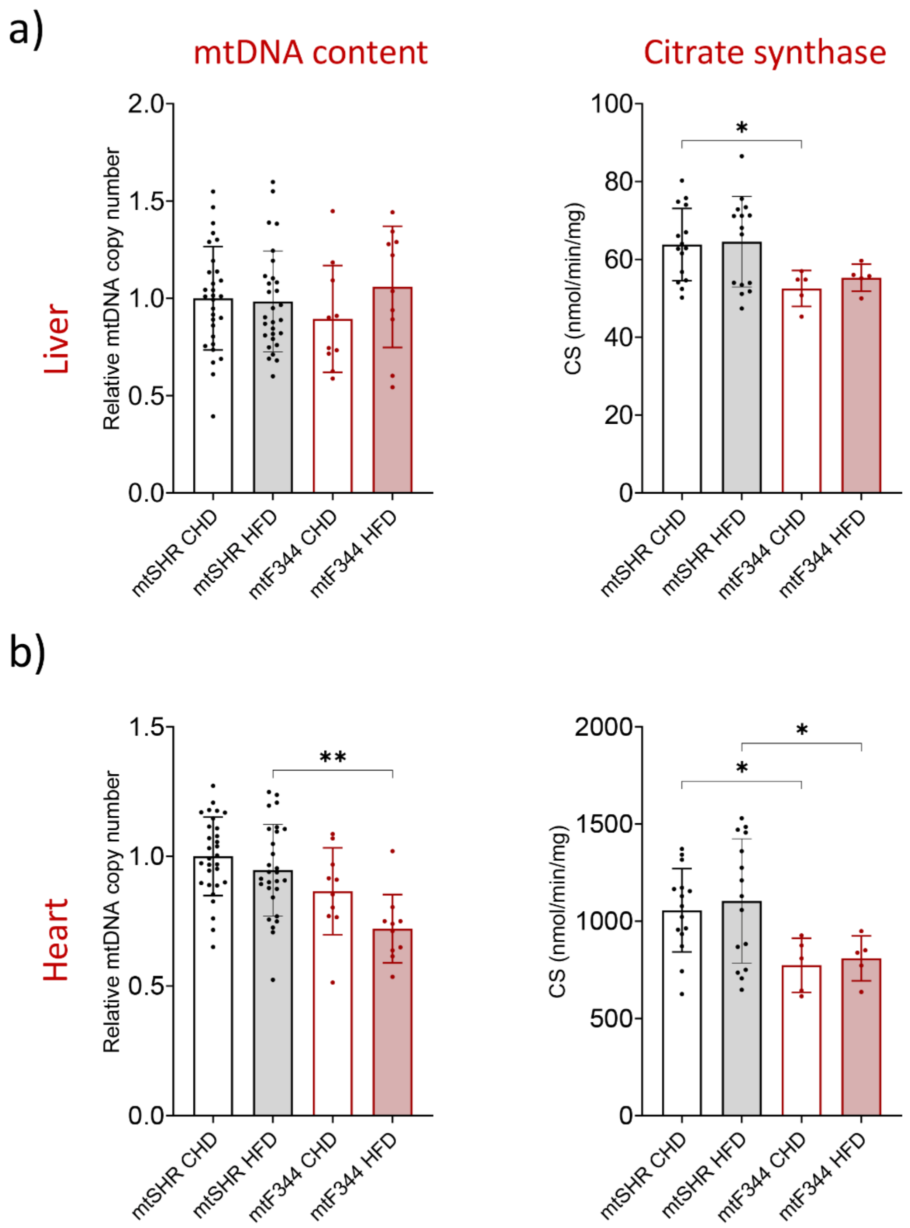

Supplementary figure 5: Content of mitochondria in mtSHR and mtF344 animals. Mitochondrial DNA copy number was measured by qPCR and citrate synthase activity (TCA cycle enzyme) was measured spectrophotometrically in liver (**a**) and heart (**b**) tissues. The data are expressed as means ± S.D. from at least 5 animals.

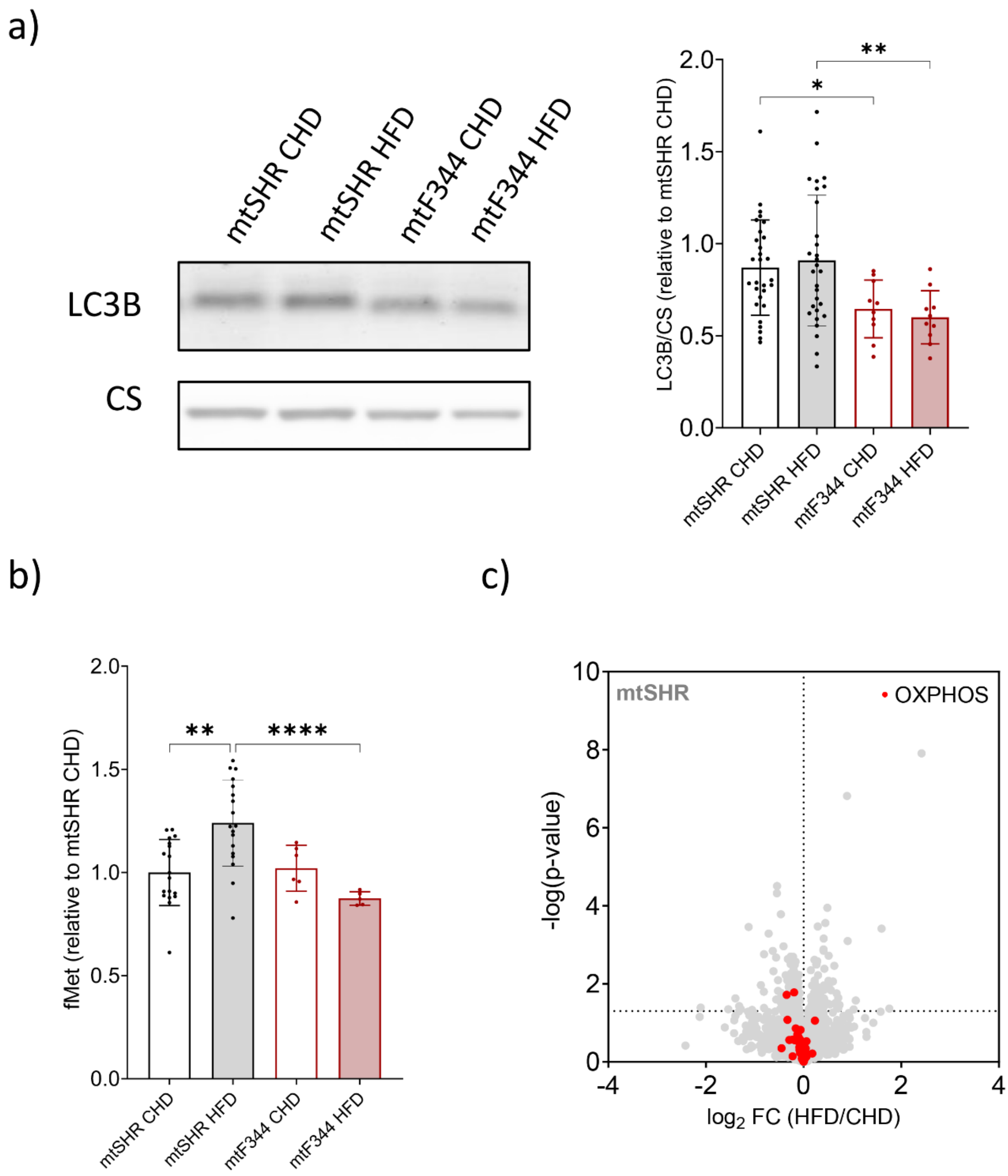

Supplementary figure 6: Analysis of autophagy and mitochondrial translation initiation in heart tissue of mtSHR and mtF344 animals. (**a**) Representative Western blot and quantification of LC3B protein in heart homogenates of at least 10 animals. Graph represents quantification of LC3B normalized to citrate synthase (CS) that was used as a marker of mitochondrial content. (**b**) The level of N-formylmethionine (fMet) in heart tissue measured by LC-MS metabolomics. The data are expressed as means ± S.D. from at least 5 animals. (**c**) Label-free quantification mass spectrometry analysis (LFQ-MS) of mtSHR heart tissue. Volcano plots depict differential contents of all proteins in between HFD and CHD. Red circles represent subunits of OXPHOS complexes. The data are expressed as means ± S.D. and each point represents the data from 6 animals.

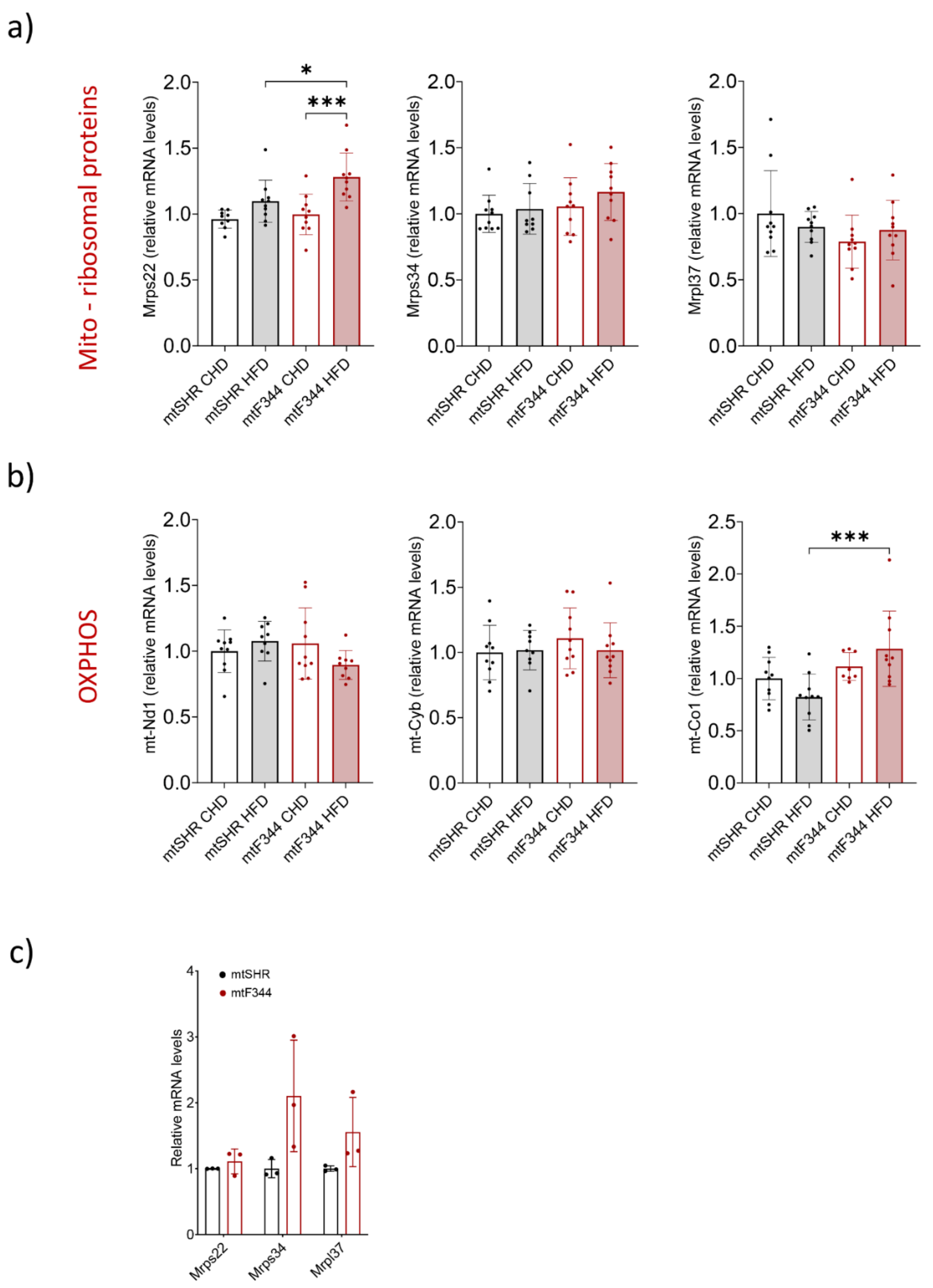

Supplementary figure 7: Relative mRNA levels of mitochondrial encoded subunits and mitochondrial ribosomal proteins. Relative mRNA levels of (**a**) mitochondrial ribosomal proteins encoded by *Mrps22* and *Mrps34* (small mitochondrial ribosomal subunit) and *Mrpl37* (large mitochondrial ribosomal subunit) genes and (**b**) OXPHOS subunits encoded by *mt-Nd1, mt-Cyb* and *mt-Co1 genes* in heart of mtF344 and mtSHR on CHD or HFD. Data represents means ± S.D. from at least 8 animals. (**c**) Relative mRNA levels of mitochondrial ribosomal proteins (as in a) in cultured skin fibroblasts derived from mtF344 and mtSHR. Data represents means ± S.D. from 3 independent expriments. Asterisks represent p-value: *<0.05; ** < 0.01; *** < 0.001; **** < 0.0001.

Supplementary table 3: SNP variants unique for SHR strain - indicative of potential heteroplasmic contamination from the founder strain (N – nucleotide).

position No.		mtSHR			mtF344			mtBN		
SHR mtDNA	RN7 reference	N	coverage (reads)	identity (%)	N	coverage (reads)	identity (%)	N	coverage (reads)	identity (%)
1137	1137	C	239	100	A	384	100	A	570	100
1521	1521	A	478	99	G	357	100	G	627	100
1585	1585	C	519	100	T	367	100	T	668	100
1716	1716	C	463	100	T	340	100	T	556	100
4351	4352	G	499	100	A	390	100	A	544	100
4695	4696	A	443	100	G	387	100	G	590	100
5198	5200	G	412	100	A	288	100	A	508	100
5200	5202	G	407	100	A	289	100	A	502	100
5235	5237	A	390	99	T	258	100	T	486	100
5267	5269	C	386	100	G	243	100	G	499	100
6976	6978	A	495	100	G	408	100	G	616	100
8019	8021	A	501	100	G	387	100	G	591	100
10225	10227	C	360	100	T	295	100	T	418	100
11223	11225	A	259	100	G	216	100	G	302	100
11358	11360	A	235	100	G	197	100	G	318	100
14773	14775	A	453	100	G	386	100	G	599	100

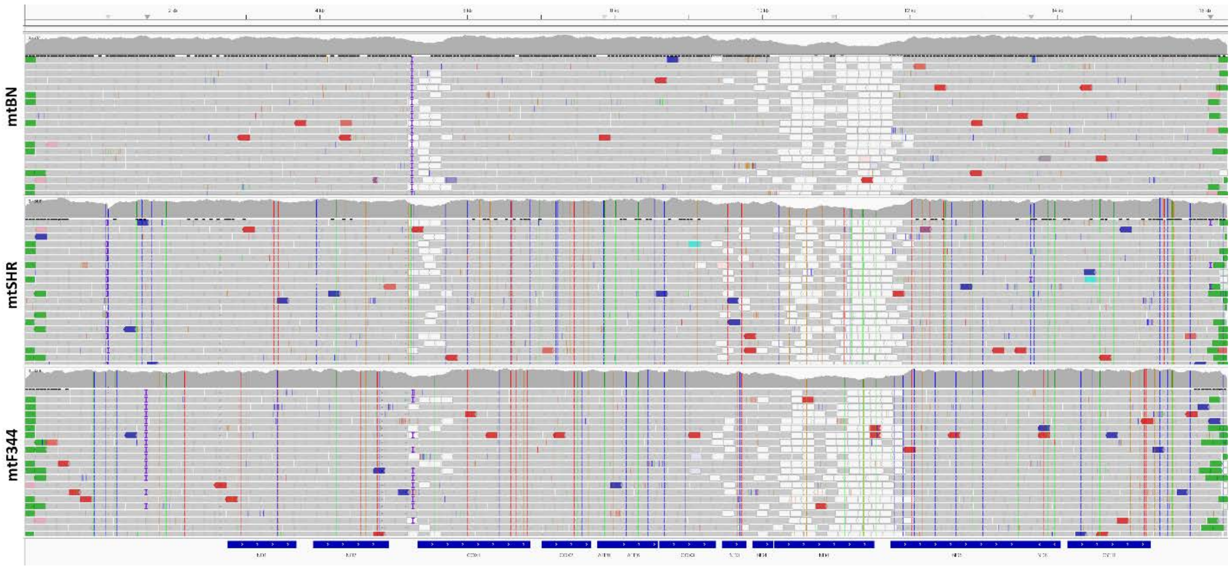

Supplementary figure 8: Mapping of individual reads for mtDNA against rn6 reference genome (BN strain based).

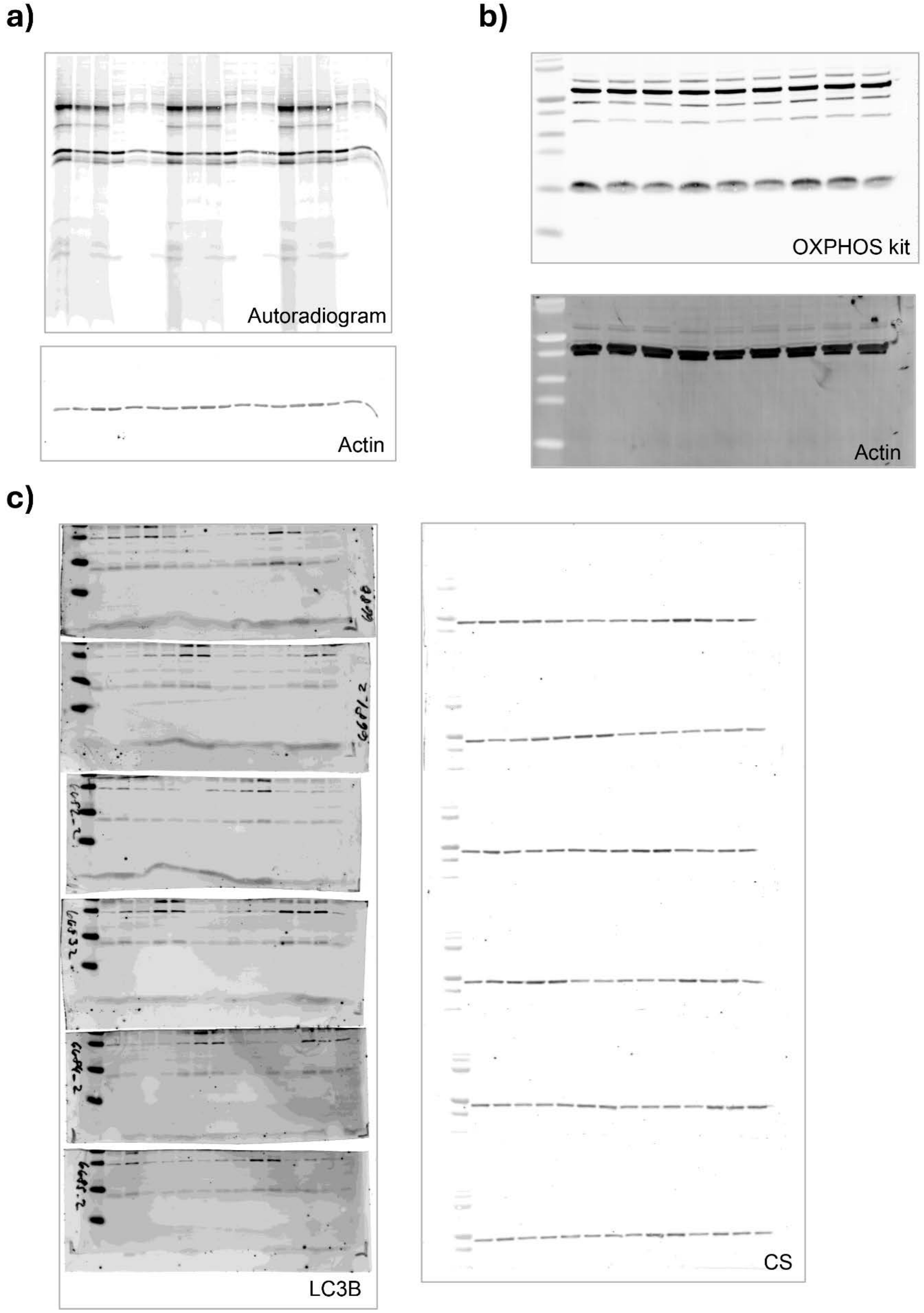

Supplementary figure 9: Uncropped and unedited Western blots images. The images were used for analyses in Figure 7a (**a**), 7c (**b**) and Supplementary figure 6a (**c**).
