# Supplementary material for: Haplotype variability in mitochondrial rRNA predisposes to metabolic syndrome
Source: Commun Biol. 2024 Sep 11;7:1116. doi: 10.1038/s42003-024-06819-w (PMC11391015; doi:10.1038/s42003-024-06819-w)
Supplement: Supplementary file 3 — Description of Additional Supplementary File [file 42003_2024_6819_MOESM3_ESM.pdf]

Description Of Additional Supplementary File

File name: Supplementary Data

Description: the source data for the graphs in the paper
