# Supplementary material for: Haplotype variability in mitochondrial rRNA predisposes to metabolic syndrome
Source: Commun Biol. 2024 Sep 11;7:1116. doi: 10.1038/s42003-024-06819-w (PMC11391015; doi:10.1038/s42003-024-06819-w)
Supplement: Supplementary file 5 — Reporting summary [file 42003_2024_6819_MOESM5_ESM.pdf]

nature portfolio

Corresponding author(s): Alena Pecinova, Tomas Mracek

Last updated by author(s): Aug 13, 2024

Reporting Summary

Nature Portfolio wishes to improve the reproducibility of the work that we publish. This form provides structure for consistency and transparency in reporting. For further information on Nature Portfolio policies, see our Editorial Policies and the Editorial Policy Checklist.

Statistics

For all statistical analyses, confirm that the following items are present in the figure legend, table legend, main text, or Methods section.

n/a	Confirmed	
☐	☒	The exact sample size (*n*) for each experimental group/condition, given as a discrete number and unit of measurement
☐	☒	A statement on whether measurements were taken from distinct samples or whether the same sample was measured repeatedly
☐	☒	The statistical test(s) used AND whether they are one- or two-sided<br>*Only common tests should be described solely by name; describe more complex techniques in the Methods section.*
☒	☐	A description of all covariates tested
☐	☒	A description of any assumptions or corrections, such as tests of normality and adjustment for multiple comparisons
☐	☒	A full description of the statistical parameters including central tendency (e.g. means) or other basic estimates (e.g. regression coefficient) AND variation (e.g. standard deviation) or associated estimates of uncertainty (e.g. confidence intervals)
☐	☒	For null hypothesis testing, the test statistic (e.g. *F*, *t*, *r*) with confidence intervals, effect sizes, degrees of freedom and *P* value noted<br>*Give P values as exact values whenever suitable.*
☒	☐	For Bayesian analysis, information on the choice of priors and Markov chain Monte Carlo settings
☒	☐	For hierarchical and complex designs, identification of the appropriate level for tests and full reporting of outcomes
☒	☐	Estimates of effect sizes (e.g. Cohen's *d*, Pearson's *r*), indicating how they were calculated

Our web collection on statistics for biologists contains articles on many of the points above.

Software and code

Policy information about availability of computer code

Data collection	n/a
Data analysis	n/a

For manuscripts utilizing custom algorithms or software that are central to the research but not yet described in published literature, software must be made available to editors and reviewers. We strongly encourage code deposition in a community repository (e.g. GitHub). See the Nature Portfolio guidelines for submitting code & software for further information.

Data

Policy information about availability of data

All manuscripts must include a data availability statement. This statement should provide the following information, where applicable:

- Accession codes, unique identifiers, or web links for publicly available datasets
- A description of any restrictions on data availability
- For clinical datasets or third party data, please ensure that the statement adheres to our policy

The numerical source data for graphs and charts are available as Supplementary data. Label-free quantification mass spectrometry (LFQ-MS) data have been deposited at PRIDE and are publicly available with accession numbers PXD045910 and PXD052976.

Human research participants

Policy information about studies involving human research participants and Sex and Gender in Research.

Reporting on sex and gender	n/a
Population characteristics	n/a
Recruitment	n/a
Ethics oversight	n/a

Note that full information on the approval of the study protocol must also be provided in the manuscript.

Field-specific reporting

Please select the one below that is the best fit for your research. If you are not sure, read the appropriate sections before making your selection.

☒ Life sciences ☐ Behavioural & social sciences ☐ Ecological, evolutionary & environmental sciences

For a reference copy of the document with all sections, see nature.com/documents/nr-reporting-summary-flat.pdf

Life sciences study design

All studies must disclose on these points even when the disclosure is negative.

Sample size	For assessment of physiological parameters at least 10 animals were used (required sample size estimated for the following parameters: difference between groups - 25%, measuring error - 20%), for other analyses at least 4 animals were used.
Data exclusions	The outlier values were excluded based on the ROUT method (Q = 2%).
Replication	Each experiment represents at least 3 biological replicates, for physiologival parameters at least 10 animals were used.
Randomization	The animals of the same genotype were randomly assigned to each experimental group. Blood and tissue collections as well as metabolomic and proteomic analyses were also randomized.
Blinding	Blinding was not performed in the study.

Reporting for specific materials, systems and methods

We require information from authors about some types of materials, experimental systems and methods used in many studies. Here, indicate whether each material, system or method listed is relevant to your study. If you are not sure if a list item applies to your research, read the appropriate section before selecting a response.

Materials & experimental systems

n/a	Involved in the study	
☐	☒	Antibodies
☐	☒	Eukaryotic cell lines
☒	☐	Palaeontology and archaeology
☐	☒	Animals and other organisms
☒	☐	Clinical data
☒	☐	Dual use research of concern

Methods

n/a	Involved in the study	
☒	☐	ChIP-seq
☒	☐	Flow cytometry
☒	☐	MRI-based neuroimaging

Antibodies

Antibodies used	Primary antibodies/cataolg #/company:<br>Total OXPHOS antibody cocktail_Mouse/ab110412/Abcam<br>Anti-LC3B_Rabbit/ab48394/Abcam<br>Anti-Citrate Synthase/ab129095/Abcam<br>Anti-Actin_ Mouse/#MAB1501/Merck<br>Anti-Actin (Ab-1) IgM_Mouse/#CP01-1EA/Calbiochem

	Secondary antibodies/cataolg #/company:<br>Alexa Fluor 680 Donkey anti-mouse IgG/#A10038/Life Technologies<br>Alexa Fluor 680 Donkey anti-rabbit IgG/#A10043/Life Technologies<br>IRDye 800 Donkey anti mouse IgG/#926-32212/LI-COR<br>IRDye 800 Goat anti-mouse IgM/#926-32280/LI-COR
Validation	All the antibodies were validated by the manufactures.<br>Total OXPHOS antibody cocktail - application - WB<br>Anti-LC3B (Abcam) - application WB, immunofluorescence<br>Anti Citrate Synthase (Abcam) - application WB, immunofluorescence, immunohistochemistry, flow cytometry<br>Anti-Actin (Merck) - application WB, immunofluorescence, immunohistochemistry<br>Anti-Actin (Calbiochem) - application WB, Immunofluorescence

Eukaryotic cell lines

Policy information about cell lines and Sex and Gender in Research

Cell line source(s)	In-house prepared primary fibroblasts from SHR and mtF344 rats.
Authentication	n/a
Mycoplasma contamination	All cell lines were negative for mycoplasma.
Commonly misidentified lines<br>(See ICLAC register)	n/a

Animals and other research organisms

Policy information about studies involving animals; ARRIVE guidelines recommended for reporting animal research, and Sex and Gender in Research

Laboratory animals	Male of inbred spontaneously hypertensive rats (SHR; SHR/OlaIpcv strain) and F344 (F344/Crl), and strains derived from SHR with the mitochondrial genome of inbred strains F344 and Brown Norway (no. of backcrossing with SHR males >50) as described in Pravenec et al., Genome Res 2007 (doi:10.1101/gr.6548207) and Houstek et al., Physiol Genomics 2014 (doi:10.1152/physiolgenomics.00069.2014).
Wild animals	n/a
Reporting on sex	Only males were used, we plan to perform the studies on females as well.
Field-collected samples	The study did not involve collected samples from the field.
Ethics oversight	Animal experiments were approved by the Institutional Animal Care and Use Committee and the Committee for Animal Protection of the Czech Academy of Sciences (Approval Number: 58/2021) in agreement with the Animal Protection Law of the Czech Republic, which is fully compatible with the guidelines of the European Community Council directives 2010/63/EU.

Note that full information on the approval of the study protocol must also be provided in the manuscript.
